# Supplementary material for: Irradiance and nutrient-dependent effects on photosynthetic electron transport in Arctic phytoplankton: A comparison of two chlorophyll fluorescence-based approaches to derive primary photochemistry
Source: PLoS One. 2021 Dec 9;16(12):e0256410. doi: 10.1371/journal.pone.0256410 (PMC8659313; doi:10.1371/journal.pone.0256410)
Supplement: S1 Table — Results of Spearman Rank correlation analyses between each underway hydrographic variable are displayed. ** is used to indicate p values < 0.001. In all instances n = 7200. (PDF) [file pone.0256410.s001.pdf]

S1 Table. Correlation of underway hydrographic variables.

	Salinity (psu)	Temp. (°C)	Chl*a* (mg m^{-3})	$\Delta O_2/Ar$
Salinity (psu)	--	0.70**	0.06**	0.09**
Temp. (°C)		--	-0.17**	-0.12**
Chl*a* (mg m^{-3})			--	0.19**
$\Delta O_2/Ar$				--

Results of Spearman Rank correlation analyses between each underway hydrographic variable are displayed. ** is used to indicate p values < 0.001. In all instances n = 7200.
