# Supplementary material for: Irradiance and nutrient-dependent effects on photosynthetic electron transport in Arctic phytoplankton: A comparison of two chlorophyll fluorescence-based approaches to derive primary photochemistry
Source: PLoS One. 2021 Dec 9;16(12):e0256410. doi: 10.1371/journal.pone.0256410 (PMC8659313; doi:10.1371/journal.pone.0256410)
Supplement: S2 Table — Sampling station locations are indicated as LS for Lancaster Sound or BS for Barrow Strait. Station mixed layer depth (MLD), mean mixed layer nitrate and nitrite concentration, and Chl a concentration within the mixed layer are shown. (PDF) [file pone.0256410.s002.pdf]

**S2 Table. CTD profiling stations**

| Location | CTD Station | Latitude (°) | Longitude (°) | MLD (m) | [$NO_3^-$ + $NO_2^-$] (μM) | Chl *a* (mg/m$^{-3}$) |
|---|---|---|---|---|---|---|
| LS | 1 | 74.316 | -80.517 | 6 | < 0.02 | 0.43 |
| LS | 2 | 74.107 | -80.511 | 9 | < 0.02 | 0.21 |
| LS | 3 | 73.987 | -80.468 | 9 | < 0.02 | 0.25 |
| LS | 4 | 75.482 | -78.643 | 8 | < 0.02 | 0.12 |
| BS | 5 | 74.222 | -93.517 | 10 | < 0.02 | 0.20 |
| BS | 6 | 74.361 | -93.556 | 6 | < 0.02 | 0.39 |
| BS | 7 | 74.599 | -93.717 | 10 | < 0.02 | 0.41 |
| LS | 8 | 74.371 | -89.621 | 9 | 0.087 | 0.50 |
| BS | 9 | 74.483 | -93.640 | 9 | 0.063 | 0.77 |
| LS | 10 | 74.486 | -80.508 | 8 | < 0.02 | 0.45 |
| LS | 11 | 74.159 | -80.470 | 6 | 0.189 | 0.24 |
| LS | 12 | 73.816 | -80.508 | 7 | < 0.02 | 0.58 |
| BS | 13 | 74.951 | -92.388 | 24 | 0.018 | 0.58 |
| BS | 14 | 75.014 | -93.115 | 14 | 0.048 | 0.28 |
| LS | 15 | 75.523 | -79.748 | 12 | 0.047 | 0.54 |
| LS | 16 | 74.234 | -86.160 | 6 | < 0.02 | 0.21 |

Sampling station locations are indicated as LS for Lancaster Sound or BS for Barrow Strait. Station mixed layer depth (MLD), mean mixed layer nitrate and nitrite concentration, and Chl *a* concentration within the mixed layer are shown.
