# Supplementary material for: Irradiance and nutrient-dependent effects on photosynthetic electron transport in Arctic phytoplankton: A comparison of two chlorophyll fluorescence-based approaches to derive primary photochemistry
Source: PLoS One. 2021 Dec 9;16(12):e0256410. doi: 10.1371/journal.pone.0256410 (PMC8659313; doi:10.1371/journal.pone.0256410)
Supplement: S3 Table — All p values were < 0.001. Standard error is reported for the regression intercept and coefficient, respectively. (PDF) [file pone.0256410.s003.pdf]

S3 Table. Surface PAR and underway photophysiological variable regression analyses

Photophysiological variable	Surface PAR	
	Lancaster Sound n = 283	Barrow Strait n = 198
F_v/F_m	$y = 0.34 - 1.4E - 4x$<br>SE = 0.0029, 8.5E-3<br>$R^2 = 0.45$	$y = 0.40 + 5.34E - 5x$<br>SE = 0.003, 0.024<br>$R^2 = 0.06$
σ_{PSII}	$y = 233.92 - 0.19x$<br>SE = 2.65, 9.6E-5<br>$R^2 = 0.64$	$y = 237.7 - 0.24x$<br>SE = 4.27, 1.8E-5<br>$R^2 = 0.42$
NPQ	$y = 0.16 - 2.4E - 4x$<br>SE = 0.006, 1.9E-5<br>$R^2 = 0.37$	$y = 0.26 - 5.7E - 4x$<br>SE = 0.009, 5.2E-5<br>$R^2 = 0.45$
$F_v/F_m\colon F_q'/F_{m'_{150}}$	$y = 0.61 + 4.7E - 4x$<br>SE = 0.006, 2.0E-5<br>$R^2 = 0.67$	$y = 0.68 + 5.0E - 4x$<br>SE = 0.0029, 4.9E-5<br>$R^2 = 0.42$
$F_q'/F_{m'_{150}}$	$y = 0.21 + 3.5E - 5x$<br>SE = 0.002, 7.4E-6<br>$R^2 = 0.07$	$y = 0.27 + 0.24E - 4x$<br>SE = 0.004, 2.5E-5<br>$R^2 = 0.37$

All p values were < 0.001. Standard error is reported for the regression intercept and coefficient, respectively.
