# Supplementary material for: Irradiance and nutrient-dependent effects on photosynthetic electron transport in Arctic phytoplankton: A comparison of two chlorophyll fluorescence-based approaches to derive primary photochemistry
Source: PLoS One. 2021 Dec 9;16(12):e0256410. doi: 10.1371/journal.pone.0256410 (PMC8659313; doi:10.1371/journal.pone.0256410)
Supplement: Attachment — Submitted filename: Response to Reviewers.pdf [file pone.0256410.s005.pdf]

University of British Columbia,
Department of Earth, Oceans, and Atmospheric Science
Vancouver, BC Canada V6T 1Z4
+1 (408)-470-0089 | ysezginer@eoas.ubc.ca

Dr. Matheus C. Carvalho
Academic Editor
PLOS ONE

Sept. 16, 2021

Dear Dr. Carvalho,

We wish to thank you for your consideration of this manuscript, the careful attention to detail apparent in comments from yourself and the Reviewers, and for the opportunity to revise. All requested changes (below) ultimately proved to be relatively minor, not changing the interpretation of the data or conclusion drawn. We provide our detailed point by point response as follows, providing further explanation where necessary. References to page and line numbers below correspond to the edited document containing tracked changes. We are confident that our responses address the insightful concerns raised, and have further improved our manuscript.

Yours Sincerely,

Yayla Sezginer

Original comments are first written in blue followed by our responses in black

Responses to the Editor

1. Please ensure that your manuscript meets PLOS ONE's style requirements, including those for file naming. The PLOS ONE style templates can be found at https://journals.plos.org/plosone/s/file?id=wjVg/PLOSOne_formatting_sample_main_body.pdf and https://journals.plos.org/plosone/s/file?id=ba62/PLOSOne_formatting_sample_title_authors_affiliations.pdf

We have amended the manuscript to meet these style and file naming conventions.

2. In your Methods section, please provide additional information regarding the permits you obtained for the work. Please ensure you have included the full name of the authority that approved the field site access and, if no permits were required, a brief statement explaining why.

Amundsen Science and the *CCGS Amundsen* were granted the following permits for our expedition.

Nunavut
Nunavut Research Institute Scientific Research License # **0501119R-M**
Department of Fisheries and Oceans License to Fish for Scientific Purposes in the waters of Nunavut # **S-19/20-1016-NU**
Parks Canada – National Parks Auyuittuq, Sirmilik and Quttirnipaaq # **ANP-2019-32477**
Canadian Wildlife Service – Access to Migratory Birds sanctuary on Bylot Island # **MM-NR-2019-NU-014**
Canadian Wildlife Service – Access to National Wildlife Areas at Coburg Island, Akpait and Qaqulluit # **NF-NR-2019-NU-008**

Foreign Waters
Vessel Clearance to conduct scientific work in Greenland waters. Danish Ministry of Foreign Affairs file # **2019-2308**
Permission for State Flight over Greenland File # **19/01017**
Government of Greenland Survey License # **G19-036**

Of these various permits, only the Nunavut Research Institute Scientific Research License # **0501119R-M** was required for the operations discussed in this manuscript. This license is now cited on page 7, line 180 in the Methods.

3. In your Methods section, please provide additional location information, including geographic coordinates for the data set if available.

The complete dataset of geographic coordinates where underway sampling took place is included in the salinity and temperature dataset available in the Polar Data Catalogue (doi: 10.5884/12715). A reference to the geographic coordinates in now provided in the study region map figure legend (Fig 1).

4. We note that you have stated that you will provide repository information for your data at acceptance. Should your manuscript be accepted for publication, we will hold it until you provide the relevant accession numbers or DOIs necessary to access your data. If you wish to make changes to your Data Availability statement, please describe these changes in your cover letter and we will update your Data Availability statement to reflect the information you provide

Salinity and temperature data collected by Amundsen Science are available in the Polar Data Catalogue (doi: 10.5884/12715). Surface photosynthetically active radiation data are available in the Polar Data Catalogue (doi: 10.5884/12518), alongside additional meteorological data provided by the Amundsen Science group of U. Laval. CTD station conductivity, temperature, and salinity data provided by the Amundsen Science group of U. Laval are available in the Polar Data Catalogue (doi: 10.5884/12713). Underway oxygen saturation and $\Delta O_2/Ar$ data are available in the Polar Data Catalogue (doi: 10.5884/13242). All Fast Repetition Rate Fluorometry measurements of phytoplankton photophysiology have been uploaded to the Polar

Data Catalogue and are currently awaiting approval. The Canadian Cryospheric Information Network reference number for this dataset is 13254, and will be trackable using this number once published. A doi will be provided as soon as available.

5. Please update your submission to use the PLOS LaTeX template. The template and more information on our requirements for LaTeX submissions can be found at http://journals.plos.org/plosone/s/latex.

We have revised the manuscript format using the PLOS LaTeX template. The reformatted manuscript and latex source code have both been uploaded under the file names Manuscript.pdf and Manuscript.tex, respectively.

6. We note that Figures 1 and 9 in your submission contain [map/satellite] images which may be copyrighted. All PLOS content is published under the Creative Commons Attribution License (CC BY 4.0), which means that the manuscript, images, and Supporting Information files will be freely available online, and any third party is permitted to access, download, copy, distribute, and use these materials in any way, even commercially, with proper attribution. For these reasons, we cannot publish previously copyrighted maps or satellite images created using proprietary data, such as Google software (Google Maps, Street View, and Earth). For more information, see our copyright guidelines: http://journals.plos.org/plosone/s/licenses-and-copyright.

Both map figures were produced in Matlab R2020a using the freely available m_map package available online at *www.eoas.ubc.ca/~rich/map.html*. The package includes a coastline, global elevation database, and river database, and provides access to publicly available bathymetry data. The data are not proprietary. A citation for m_map has been added to the map figure legend and the references section (Fig 1).

Responses to Reviewer 1:

"The major point is that there is no support of apparent (measured) O2 evolution and C assimilation rate for the two ETRs in the study area. The amplitude based ETRa have been developed as biophysical models to derive gross primary production (GPP) because FRRf can reduces only QA but not PQ pool and thus examine the electron flow rate for oxygen evolution. However, ETRa-based estimated GPP does not always equal to apparent GPP (Regaudie-de-Gioux et al., 2014). Many previous studies have investigated the factor affecting the relationships between modeled ETRa and measured O2 evolution rate (Suggett et al., 2001; Robinson et al., 2009; Deblois, Marchand & Juneau, 2013) and C assimilation rate in natural communities (Lawrenz et al., 2013; Schuback et al., 2017; Zhu et al., 2017; Hughes et al., 2018b; Ryan-Keogh et al., 2018; Kazama et al., 2021). For oxygen-based GPP, it is well confirmed for cultivated species (Suggett et al., 2009) but species composition can affect conversion factor in natural communities (Deblois et al., 2013). For, C-based GPP, although there are many studies but no global model is derived to convert ETRa to GPP (Hughes et al., 2018a). On the other hand, authors' models is not supported by oxygen production rate or C assimilation of algae yet. The kinetic based ETRk by FIRe system (FIRe-ETRk) is a novel method to improve the errors from the parameters (Gorbunov & Falkowski, 2021). The relationships between the FIRe-ETRk and

the growth rate (net primary production, NPP) of two model species, Thalassiosira pseudonana, and Dunaliella tertiolecta were examined, but not in natural community yet. Also the relationships between FIRe-ETRk and GPP is not examined yet. Therefore, the use of authors' kinetic model in natural algal communities without reference measured productivity (O2 or C) must be considered carefully, even if it is likely analogous to the FIRe-ETRk.

Because authors' models is not supported by oxygen production rate of algae yet, the absolute value of ETRk cannot compare to ETRa. For example, when these two ETR values are used as estimators of GPP and NPP, respectively, in the study area, the results are still questionable. Because net productivity does not include respiration, ETRk must be lower than ETRa on the same phytoplankton assemblage. However, present study clearly showed ETRk > ETRa in Lancaster Sound (Fig. 8). This paradoxical results may be due to underestimation of ETRa, and/or overestimation of ETRk. The former is likely due to inadequate dark acclimation time (5 min, P11 L211). For rapid light curve, there is no consensus rule but typically 10~20 min of dark adaptation period is used (Schuback et al., 2021).The latter is likely due to the underestimation of turnover rate, as authors pointed out. If authors primarily focus to the comparison of relative oxygen productivity between Lancaster Sound and Barrow Strait, use Fv/Fm and ETRa but not ETRk."

The Reviewer makes excellent points but appears to have mis-interpreted the aims and scope of our study. We agree that electron transport rates (ETR) are not equivalent to oxygen evolution or carbon uptake. Indeed, as noted by the reviewer, there are many photosynthetic and metabolic processes that can lead to the decoupling of electron transport from oxygen evolution and downstream carbon assimilation. However, it was not our intention in this article to use ETR as an absolute metric of primary productivity. Rather, as we state in the introduction (P6 L150), our goal is to compare different approaches to estimating ETR, examining how the two contrasting algorithms perform across a range of hydrographic regimes. As pointed out by the Reviewer, there is a significant need for work of this kind in natural environments, moving beyond current results that have focused on laboratory studies. Even without 'calibration' against independent productivity bench-marks (O2 evolution, CO2 uptake), ETR is an important diagnostic of photosynthetic capacity in its own right. The reducing power produced by this process fuels a number of metabolic functions, and presents an upper limit for carbon fixation by the Calvin-Benson cycle. We understand that the comments of the reviewer may have reflected a lack of clarity in our initial wording. We therefore have altered our wording throughout from 'primary productivity' to 'primary photochemistry' to remove any suggestion that ETR is interchangeable with carbon uptake or oxygen evolution. We wholeheartedly agree that validating ETR measurements with parallel measures of O_2 evolution would enable us to assess the accuracy of the different ETR algorithms. This has now been further emphasized in the discussion section, where we suggest such work for a follow up study (P30 L862).

The reviewer also raises some concerns about the effects of respiration on our results, suggesting that ETRk and ETRa measure NPP and GPP, respectively. This is, in fact, incorrect, as both ETR algorithms provide estimates of GPP. The kinetic ETR algorithm recently developed by Gorbunov and Falkowski (2021) evaluates the rate of electron transport through Photosystem II (PSII) as the normalized photochemical yield at a given light intensity multiplied

by the rate at which the primary electron acceptor, Q_a is capable of turning over electrons from the photosynthetic Reaction Center II to the secondary electron acceptor, Q_b. As such, ETR_k measures primary photochemistry and is theoretically equivalent to ETR_a, which measures the rate of electron transport through PS II as the product of the photochemical energy conversion efficiency, the functional light absorption area of PSII, and the provided light intensity. Although Gorbunov and Falkowski (2021) used the high correlation between lab-grown phytoplankton growth rates and ETR_k to validate their novel kinetic-based ETR algorithm, ETR_k is not a measure of NPP, which is the difference between GPP and respiration.

Minor points

P2 L25 fast repetition rate fluorometry

No change, we prefer to keep the capitalization for consistency with the literature.

P5 L93 photosynthetically active radiation

Resolved. (P5 L106)

P8 L157 Provide company name and city for every instruments of the manuscript.

Provided company name where missing. Did not include city name, to keep with style of previous PLOS One publications (see Schuback et al., 2015)

P8 L178 Unify the unit of whole manuscript (s, min).

No change (unless the Editor and Editorial process requires) since our results capture processes occurring on differing time-scales, we prefer to use different units of time, as appropriate.

P10 L207 Provide the version and company of the software.

Resolved company name. Software version/name provided.

P10 L209 Table 1 What is the ChlF?

Replaced with Chl*a*

Å² PSII−1, Definition of Fq'/Fm' (max) should not be "under 150 μmol quanta m-2 s-1".

Resolved.

P11 L217 Provide the duration of each acquisitions and total time per sample.

Resolved. P10 L253

P11 L218 Use mol instead of E.

Resolved.

P11 L226 Provide the duration of each light step. For rapid light curve, less than 30 s is recommended (Perkins et al., 2010).

Resolved. P10 L260

P13 L254-258 Å² PSII−1

Resolved.

P13 L262 Provide the reference for 3 component multi-exponential model.

Resolved. P13 L325

Equation (2) What are the F(t) and CQA(t)?

Resolved. Measured fluorescence and fraction of RCIIs closed by excitation flashlets. P14 L345

P13 L273-276 QA, QB

Set to Q_a and Q_b throughout, removed all instances of QA and QB

P16 L319 Provide version, company and city of the software.

Added company. Version included. (P13 L342)

P17 L349 Table 2 Correct notation Chl a.

Resolved. Table 2 moved to the supplements.
P17 L354 Table 3 Does the zero mean true zero or lower than detection limit?
(mg m−3)
Zeros do indicate below detection limit. Values below detection limit in have been updated to < 0.02 uM. Table 3 has been moved to the Supporting Information section (S2 Table)
P19 L387 Spearman's rank correlation. Use ρ (rho) or "rs" instead of R. See Schober et al. 2018. Correlation Coefficients: Appropriate Use and Interpretation, Anesthesia & Analgesia: Volume 126, 5, 1763-1768. doi: 10.1213/ANE.0000000000002864
Resolved throughout.
P20 L403 Use 25th and 75th percentile, or range with median value, instead of SD.
Deviation from the median reported is the median absolute deviation, not the SD.
P20 L410-415 Integrate this part into discussion.
We prefer to retain this aspect in the results, as these lines provide a quick operational note, rather than a discussion of key findings.
P20 L417 Explanation is needed why 150 μmol quanta m-2 s-1 is used.
Explanation added to Methods (P11 L283)
P21 L423-426 Integrate this part into discussion.
Agreed. We have moved the majority of text outlining our interpretations of Fig 5 to P24 L672-678 in the Discussion section.
P22 L460 Fig. 8
Resolved
P23 L485 Spatial variation of photophysiology?
Adopted suggestion. (P22 L609)
P24 L512 Provide the reference of "model predictions".
Resolved. (P23 L637)
P24 L517 Correct notation Fv/Fm as in Table 1.
Resolved throughout.
P28 L598-625 These paragraphs should be included in Methods
After some consideration, we are confident that these paragraphs belong in the discussion, since they provide critical context to understand how differing numerical procedures may affect results. Further, these lines include a description of an analogous method that we did not apply - including this in the Methods could lead to some confusion in what we actually do, as opposed to what we contrast against.
P32 L683- Follow the style in journal guidelines.
Resolved as per Editor's comments.

Responses to Reviewer 2:

"The analysis is, to my knowledge sound and robust, but I have some issues with the narrative. I think this stems to some extent to the introduction not really clearly presenting the authors' hypotheses. I don't think all science needs to be hypothesis-based but here we can identify several research questions but I don't feel I got a good sense of what was anticipated and why. The end of the introduction would be where I would expect to find this, rather than the recap of key methods/results/discussion in this iteration. I would also recommend highlighting the biggest finding in the title instead of a general description of what was done. The title had me looking forwards to the "assemblages" part but since it wasn't possible to address community

composition in any way with this dataset I wonder if there isn't a more just way to phrase this.

Another question that I would like to raise relates to the relationship between photochemical efficiency, environmental stress and species composition. In my experience, using lab cultures, fluorescence can vary a lot between taxonomic groups despite ressource replete and exponential growth conditions (notably I'd consider FvFm values to be higher for diatoms and greens and owcr for (pico)cyanobacteria and heterotrophs). I guess I really feel like knowledge of species composition at lower resolution is necessary at this point to distinguish whether we are measuring stress or community changes as a result of changes in the environment (including stress!). Although I don't think there is a whole lot of literature discussing this, I would be happy to see some support to whatever position is taken.

Finally I would have been interested in knowing how high resolution the data needs to be to have adequate dark acclimation. How many fewer samples would be analyzed with a longer dark acclimation? (10, 20, 30+ min) and how would this affect the results (maybe this adds too much to the text but comparing the statistical power of the tests used and by subsampling the current set based on different scenarioswould be one way to elegantly present this...).

If PLOS ONE does supplementary material: Do the hydrographic variables for each station need to be presented in the main text? This data is great to make available but I might stick to presenting the data directly relevant to the analyses (graphically to highlight differences between LS and BS) and leave the rest out of the main text."

The Reviewer raises important points that we have addressed:

1. We have added our research question and objective to the introductory paragraph (P6 L150), and refined our title, as suggested, to ensure more transparency in what was anticipated and why.
2. We agree entirely with the Reviewers point, and have shown previously from borad analysis of numerous cultures (e.g. Suggett et al. 2009) that diatoms and greens result in very different inherent FRRf parameterization (e.g. higher Fv/Fm) than pico and nano phytoplankton, but also that these trends are hard to deconvolve from patterns of resource availability for photosynthesis. We therefore argue that variability in Fv/Fm results from both direct physiological effects of nutrient limitation, and indirect effects that increase the abundance of smaller phytoplankton taxa (with typically lower Fv/Fm) in nutrient limited environments. We make this point on P24 L680-714, and acknowledge the unfortunate lack of species composition data in our study to fully resolve this issue.
3. We recently conducted additional experiments to address the question of dark acclimation time-scales. (These experiments were conducted this past year, i.e. on a different cruise from that presented in this paper). We allowed samples to dark acclimate over 30 minutes and took FRRf measurements every 2 minutes to assess F_v/F_m and σ_{PSII} recovery throughout the course of the dark acclimation period. Samples required a mean recovery time of 9.65 ± 1.53 min (n = 34) to reach stable, maximum light absorption efficiencies. The sampling approach used in this current study (2019 data) used a 5 min acclimation time. Increasing this to 10 minutes would increase the overall sampling interval to 17 minutes, which would have reduced our sample size from 481 to 340,

representing a 29.5% reduction in measurement resolution. These preliminary results have been added to the Discussion section (P26 L749-764).

4. We prefer to keep the hydrographic data in the main text to provide context for the physical properties of our sampling region. We have moved the table of correlation statistics between hydrographic variables to the supplemental section, as we agree that it is not critical to the narrative presented. All the hydrographic data is publicly available on the Polar Data Catalogue (doi: 10.5884/12715).

Detailed comments:
Make sure the tables and figures are presented in order of appearance (I think Fig 8 is mentioned very early on). Spell check the tables.
Resolved.
line 318: Is it necessary to say data wasn't corrected for autocorrelation? Otherwise specify why this wasn't necessary.
We have removed this line.
Figures are a bit fuzzy...can this be improved?
We have reformatted the figures according to PLOS One specifications
Fig1: Should have insert of greater geographical scope to better situate sites
Resolved.
Fig2 and 3: grey highlight should be under curve
Resolved.
Fig 3: match axis colors to the colors of the curve? Improve axis clarity (put long labels on 2 lines?)
Resolved.
Fig4 and 5: If possible add confidence interval? Why weren't the rank correlations calculated for BS and LS separately if we are interested in comparing these two locales
Resolved. Computing separate correlations for BS and LS has led to some new discussion of the differences between these two regions, particularly regarding $Fq'/Fm'_{(150)}$ and PAR relationship. We interpret the positive relationship between $Fq'/Fm'_{(150)}$ and PAR in Barrow Strait as an indicator of the increased ability of phytoplankton in Barrow Strait to acclimate to higher light levels. In contrast, the lack of a relationship between $Fq'/Fm'_{(150)}$ and PAR in Lancaster Sound is further evidence of possible photodamage to reaction centers, likely due to insufficient N required to repair non-functional reaction centers. We discuss these results on P24 L672-678.

Fig 7: Fv/Fm seems related to the relationship between ETRk and ETRa...except for the steepest slope of points which has very high Fv/Fm values. This is presented in the results but isn't discussed as far as I can tell. I would be curious to know more about why this site is different.
The unusual site where Fv/Fm is very high, but has a large decoupling ratio between ETR_k:ETR_a is unique in that it has a very low Fq'/Fm'_{emax} value (< 0.04) despite its high Fv/Fm value (> 0.42). Fv/Fm and Fq'/Fm'_{emax} are highly correlated ($\rho = 0.93$ in our dataset), so it is likely this site is an outlier given we have one of our highest Fv/Fm and lowest Fq'/Fm'_{emax} values here. This explanation is now given on P27 L792-809.

Fig8: hard to interpret. Could sites close by be averaged to better distinguish between day/night sites? Which could then be kept of similar size?

After spending some time rethinking how to best represent the data presented in Fig 9 (we believe Reviewer 2 is actually commenting on Fig 9), we've resolved to keep the figure as is. We believe that averaging samples across broad areas would obscure small-scale features in photophysiological spatial variability, which we believe is one of the unique and valuable aspects of our study.

Responses to Reviewer 3:

"One concern I have with the paper is the use of the term "primary productivity" throughout the manuscript when speaking of the FRRf measurements. Generally (though not exclusively), this term is used to refer to either oxygen production or carbon fixation. While electron transport rates are correlated with these, they are not synonymous with them. As the authors are very well aware given their previous work, the difficulty involved in converting measurements of electron transport rates to carbon fixation has been the major stumbling block to utilizing shipboard FRRf measurements to estimate productivity in oceanographic studies. Thus, the use of the term primary production to refer to electron transport measurements derived from either of the two ETR algorithms is misleading. This is particularly so given that no effort is made in the study to directly measure photosynthetic oxygen production or carbon fixation or to determine which of the two algorithms provides a better estimate of either (as the authors point out, this is important for future work). As such, I believe it would be better to use terms like "electron transport rates" or "primary photochemistry" when referring to the measurements derived from the FRRf."

In agreement also with Reviewer 1's first comment, we have adopted the Reviewer 3's suggestion of switching our language from 'primary productivity' to 'primary photochemistry' to avoid confusion with carbon or oxygen-based productivity measures.

Line 261: Change "ETRk" to "ETRk".
Resolved.
Line 309: There are several instances in the paper where Fv/Fm is not italicized. Please make sure that italics are consistently used.
Resolved.
Line 460: Change "Fig8" to "Fig 8".
Resolved.
Line 487: This paragraph could be cleaned up slightly. Specifically, the sentence in line 500, "we infer that nitrogen, rather than iron deficiency was the most likely cause of low photo-efficiencies", is something of a repeat of the sentence at line 493, "suggest that the low Fv/Fm values we observed likely reflect nitrogen deficiency."
Removed repetitive statements.
Line 584: Change "F'q/F'm" to "Fq'/Fm' ".
Resolved
Line 591: Change "nutrient limiting" to "nutrient-limiting".
Resolved.
Line 657: Change "NQP" to "NPQ".
Resolved.
Line 660: Change "F'q/F'm" to "Fq'/Fm' ".

Resolved.
Figure 3: The y-axis label for Figure E (Fq'/Fm' 150) is somewhat confusing to read. The 150 is in line with the label for Figure D and at first it was unclear which figure it belonged to. Adjusting the figure so that the labels are not in line with or so close to each other would make it easier to read.
Resolved.
Figure 4: correct uE to μE in x-axis labels.
Resolved.

Once again, we thank each of the Reviewers for their insightful questions and comments, which we feel have enhanced the quality of this manuscript.
