# Supplementary material for: Irradiance and nutrient-dependent effects on photosynthetic electron transport in Arctic phytoplankton: A comparison of two chlorophyll fluorescence-based approaches to derive primary photochemistry
Source: PLoS One. 2021 Dec 9;16(12):e0256410. doi: 10.1371/journal.pone.0256410 (PMC8659313; doi:10.1371/journal.pone.0256410)
Supplement: Attachment — Submitted filename: PONE-D-21-24983_Review.docx [file pone.0256410.s004.docx]

Review of PLOS ONE

Chlorophyll fluorescence-based estimates of photosynthetic electron transport in Arctic

phytoplankton assemblages	Aug 29 2021

General comments

The analysis is, to my knowledge sound and robust, but I have some issues with the narrative. I think this stems to some extent to the introduction not really clearly presenting the authors’ hypotheses. I don’t think all science needs to be hypothesis-based but here we can identify several research questions but I don’t feel I got a good sense of what was anticipated and why. The end of the introduction would be where I would expect to find this, rather than the recap of key methods/results/discussion in this iteration. I would also recommend highlighting the biggest finding in the title instead of a general description of what was done. The title had me looking forwards to the “assemblages” part but since it wasn’t possible to address community composition in any way with this dataset I wonder if there isn’t a more just way to phrase this.

Another question that I would like to raise relates to the relationship between photochemical efficiency, environmental stress and species composition. In my experience, using lab cultures, fluorescence can vary a lot between taxonomic groups despite ressource replete and exponential growth conditions (notably I’d consider FvFm values to be higher for diatoms and greens and ower for (pico)cyanobacteria and heterotrophs). I guess I really feel like knowledge of species composition at lower resolution is necessary at this point to distinguish whether we are measuring stress or community changes as a result of changes in the environment (including stress!). Although I don’t think there is a whole lot of literature discussing this, I would be happy to see some support to whatever position is taken.

Finally I would have been interested in knowing how high resolution the data needs to be to have adequate dark acclimation. How many fewer samples would be analyzed with a longer dark acclimation? (10, 20, 30+ min) and how would this affect the results (maybe this adds too much to the text but comparing the statistical power of the tests used and by subsampling the current set based on different scenarioswould be one way to elegantly present this...).

If PLOS ONE does supplementary material: Do the hydrographic variables for each station need to be presented in the main text? This data is great to make available but I might stick to presenting the data directly relevant to the analyses (graphically to highlight differences between LS and BS) and leave the rest out of the main text.

Detailed comments:

Make sure the tables and figures are presented in order of appearance (I think Fig 8 is mentionned very early on). Spell check the tables.

line 318: Is it necessary to say data wasn’t corrected for autocorrelation? Otherwise specify why this wasn’t necessary.

Figures are a bit fuzzy...can this be improved?

Fig1: Should have insert of greater geographical scope to better situate sites

Fig2 and 3: grey highlight should be under curve

Fig 3: match axis colors to the colors of the curve? Improve axis clarity (put long labels on 2 lines?)

Fig4 and 5: If possible add confidence interval? Why weren’t the rank correlations calculated for BS and LS separately if we are interested in comparing these two locales

Fig 7: Fv/Fm seems related to the relationship between ETRk and ETRa...except for the steepest slope of points which has very high Fv/Fm values. This is presented in the results but isn’t discussed as far as I can tell. I would be curious to know more about why this site is different.

Fig8: hard to interpret. Could sites close by be averaged to better distinguish between day/night sites? Which could then be kept of similar size?
